# Charcoal Hemoperfusion for Methotrexate Toxicity: A Safe and Effective Life-Rescue Alternative When Glucarpidase Is Not Available

**DOI:** 10.3389/fped.2021.635152

**Published:** 2021-08-19

**Authors:** Alejandra Rosales, Alvaro Madrid, Marina Muñoz, Jose Luis Dapena, Gema Ariceta

**Affiliations:** ^1^Department of Pediatrics, Medical University Innsbruck, Innsbruck, Austria; ^2^Pediatric Nephrology, University Hospital Sant Joan de Deu, University of Barcelona, Barcelona, Spain; ^3^Pediatric Nephrology, Vall d'Hebron Hospital, Universitat Autònoma de Barcelona, Barcelona, Spain; ^4^Pediatric Oncology and Hematology Department, Vall d'Hebron Hospital, Universitat Autònoma de Barcelona, Barcelona, Spain; ^5^Pediatric Oncology and Hematology Department, University Hospital Sant Joan de Deu, University of Barcelona, Barcelona, Spain

**Keywords:** charcoal, hemoperfusion, methotrexate, high dose methotrexate, glucarpidase, methotrexate toxicity

## Abstract

**Background:** High dose methotrexate (HDMTX) is used for the treatment of pediatric hemato-oncological diseases. HDMTX can induce acute kidney injury in cases of delayed elimination. The use of leucovorin remains the most effective rescue action. Further treatment options are of difficult access in the rare cases where leucovorin fails to prevent renal failure from occurring. Glucarpidase is an effective treatment in cases of methotrexate (MTX) delayed elimination, but cost is high and availability is limited. Charcoal hemoperfusion (CHP) is a very efficient procedure to remove protein-bound drugs, promoting fast MTX elimination, but is rarely considered as a treatment option.

**Methods:** We present three pediatric cases with prolonged exposure to MTX after HDMTX and delayed elimination in which hemoperfusion was performed as rescue treatment for methotrexate intoxication.

**Results:** Charcoal hemoperfusion was performed with positive results and no complications as bridging until glucarpidase was available in two cases and in one case where two doses of glucarpidase led to insufficient reduction of MTX levels.

**Conclusions:** CHP can be considered as a rescue treatment option in MTX intoxication, since it is an effective and safe extracorporeal method for removing MTX, in cases where rescue with leucovorin is insufficient and glucarpidase is not available or while waiting for delivery.

## Introduction

Methotrexate (MTX) is administered in high doses (HDMTX, dose > 500 mg/m^2^) for the treatment of several hemato-oncological diseases in the pediatric age (including lymphoblastic leukemia, lymphoma and osteosarcoma). MTX is a folate analog antimetabolite that interferes with folate metabolism impeding purine and thymidine synthesis and DNA production promoting cell death. MTX action is cell-cycle dependent, acting specifically during DNA-synthesis and making tissues with high turnover more susceptible to cytotoxicity. Fifty percent of circulating MTX is bound to proteins, mostly albumin, regardless of its concentration in serum. MTX molecules are 454 Da, and MTX has a distribution volume of 0.4/0.8 L/kg (1, 2). Elimination occurs through glomerular filtration and tubular secretion. At a urinary pH <7, MTX and its metabolites precipitate in renal tubules leading to delayed elimination and prolonged exposure times, causing further MTX precipitation and perpetuating renal damage (3).

HDMTX regimens include intensive hydration and urine alkalinization to prevent acute kidney injury (4, 5). Monitoring of plasma MTX levels, monitoring diuresis and urinary pH are mandatory to prevent toxicity. Nevertheless, inadequately high MTX levels are observed in 0.5–1.8% patients, increasing the risk of MTX toxicity (4, 6, 7). Factors such as impaired renal function, presence of ascites, pleural effusion or bowel obstruction, or concomitant administration of certain drugs (such as antibiotics, aspirin, probenecid, and proton pump inhibitors), increase the risk of MTX toxicity (4). Prolonged exposure to MTX results not only in renal impairment but also in gastrointestinal distress, hepatic insufficiency, and bone marrow suppression (8).

Leucovorin (folinic acid) competes with MTX and prevents this drug toxicity in normal cells: it provides reduced folates to bypass the metabolic blockage produced by MTX (9). Leucovorin remains one of the cornerstones of preventing MTX toxicity, since it is the only available approach that acts intracellularly. Treatment protocols with HDMTX include a 2–3 days period of multiple leucovorin doses. Safety of HDMTX regimens requires careful monitoring of blood MTX levels for the adjustment of leucovorin doses. Hydration and alkalinization should be continued and adjusted, since successful rescue with leucovorin depends on patient renal elimination of MTX (10).

Glucarpidase (Voraxaze™, a recombinant bacterial carboxypeptidase G2) is a very effective treatment option for MTX poisoning. Glucarpidase provides an enzymatic method for MTX cleavage into non-toxic metabolites, 2, 4-diamino-N(10)-methylpteroic acid (DAMPA) and glutamate. A single dose of glucarpidase can reduce MTX level by >95% in 15 min, but it has no influence in intracellular MTX levels. Cost is elevated and availability limited (in the European Union only on compassionate use) often causing delays in the start of treatment (10).

Diverse reports exist on the use of extracorporeal treatments for MTX intoxication. The choice of a treatment method for drug removal depends on molecular weight of the drug, protein binding, volume of distribution, treatment availability, and expertise in the center. Hemodialysis is a suitable method for low molecular weight, water soluble molecules. Larger size molecules, such as MTX, are insufficiently removed by standard HD and require high flux filters (11). High-flux hemodialysis is the most frequently implemented extracorporeal therapy in cases of MTX toxicity but efficacy is limited (12). MTX redistribution from the cellular compartment or third space may result in a rebound of MTX levels after treatment discontinuation, requiring repeated dialysis or CHP sessions (10).

Charcoal hemoperfusion (CHP) is a successful extracorporeal method for the removal of a variety of toxins. It is a suitable method for toxins with high molecular weight, a low volume of distribution and high protein binding, where hemodialysis is less effective. Table 1 shows previous reports on the use of CHP in cases of MTX toxicity alone or combined with other extracorporeal treatments and they results (Table 1). We present three pediatric cases in which CHP was used successfully for the treatment of MTX intoxication when glucarpidase was not available.

**Table 1:** Existing reports on the use of hemoperfusion in MTX toxicity cases.

**References**	**Case number (age, gender)**	**Indication (treatment protocol)**	**MTX dose**	**Supportive treatment**	**Extracorporeal method**	**Results**
Djerassi et al. (13)	4 (?)	Osteosarcoma	300 mg/kg	Citrovorum Factor	CHP and/or iHD	CHP more effective than IHD at low MTX levels
Gibson et al. (14)	1 (56 y, f)	Breast carcinoma	600 mg	Leucovorin	HP (Amberlite XAD-4 Column)	Amberlite XAD-4 hemoperfusion effective Charcoal more effective than XAD-4 *in vitro* Rebound after treatment observed, no sustained effect
Bouffet et al. (15)	3 (71, 7, 52 y)	?	1.5 and 3 g/m^2^	?	CHP, iHD, PE	CHP efficient, no complications observed Plasma exchange less effective HD did not decrease MTX levels but corrected renal failure.
Molina et al. (16)	1 (60 y, m)	Lymphocytic lymphoma	130 mg/m^2^	Allopurinol, urine alkalinization, citrovorum	iHD + CHP	Sustained reduction of MTX levels using sequential HD and CHP.
Relling et al. (17)	1 (15 y, f)	Osteosarcoma (OS-86)	(12 g/m^2^)	Hydration, urine alkalinization, Leucovorin, Thymidine	iHD + CHP Thymidine	Combined HD-CHP more effective than HP only.
Grimes et al. (18)	1 (18 y, f)	Osteosarcoma	8 g/m^2^	Urine alkalinization, leucovorin, oral activated charcoal	CHP, iHD	Rebound of MTX levels after CHP and HD. Authors attribute more success to supportive treatment than extracorporeal therapies.
McIvor (19)	1 (39 y, m)	Burkitt Lymphoma	3 g/m^2^	Allopurinol, urine alkalinization, leucovorin	CHP	Rapid reduction of MTX level after CHP. No complications.
Nowicki et al. (20)	1 (10 y, m)	Osteosarcoma	12 g/m^2^	Hydration, urine alkalinization, leucovorin	iHD and CHP, glucarpidase	HD due to hyperkalemia, followed by CHP. Significant reduction of MTX levels using HD, HP and glucarpidase.
Nemoto et al. (21)	1 (12 y, f)	Osteosarcoma	10 g/m^2^	Hydration, urine alkalinization, leucovorin	HP, iHD and PE	Effective removal of MTX using CHP. HDF due to renal failure. PE due to liver failure. Significant reduction in MTX level after CHP, no significant reduction after PE
Grafft et al. (22)	1 (64 y, f)	B-cell lymphoma	8 g/m^2^	Hydration, urine alkalinization, leucovorin	CHP and high dose CVVH	CVVH combined with CHP not more effective than CVVH alone.
Chan and Hui (11)	1 (11 y, f)	Osteosarcoma (HKPSOSG)	12 g/m^2^	Hydration, urine alkalinization, leucovorin	SPAD + CHP	Faster drop in MTX level after sequential use of SPAD and CHP than using only leucovorin

## Materials and Methods

Three pediatric cases which presented delayed MTX elimination after HDMTX during the treatment of different oncological diseases are presented in this report. The clinical and laboratory data were analyzed retrospectively. Details on treatment protocol, definition of toxic range and leucovorin rescue are presented in Table 2.

**Table 2:** Detailed information on treatment protocols.

**Study, References**	**MTX dose**	**Duration of infusion**	**Hyperhydration**	**Leucovorin**	**Time to first leucovorin**	**MTX at 24 h**
EURAMOS (23)	12 g/m^2^	4 h	3,000 ml/m^2^	15 mg/m^2^, adjusted to nomogram	24 h	<8.5 μmol/L
Euro LB 02 (24)	5 g/m^2^	24 h	3,000 ml/m^2^	15 mg/m^2^	42 h	<150 μmol/L
ALL SEHOP/PETHEMA 2013 HR (25)	5 g/m^2^	24 h	3,000 ml/m^2^	15 mg/m^2^	42 h	<150 μmol/L

MTX levels were measured using the Architect Methotrexate chemiluminescent assay (Abbott Diagnostics, IL, USA) on the Architect i2000SR (Abbott Diagnostics). Plasma MTX levels may be overestimated in this report, since the method measures not only MTX but also its metabolites due to antibody cross reactivity.

Hemoperfusion was performed using the Prismaflex® system CHP with Adsorba® C 300 kit (cellulose-coated activated charcoal). The procedure required a central venous access using a dual-lumen hemodialysis catheter. Anticoagulation was performed with heparin. Sessions lasted 2.5–3 h.

All the procedures being performed were part of the routine care and were performed in accordance to relevant guidelines and regulations. In view of the retrospective nature of the study, the need for informed consent was waived by the local ethics committee.

## Results

### Patient 1

**Figure 1:**
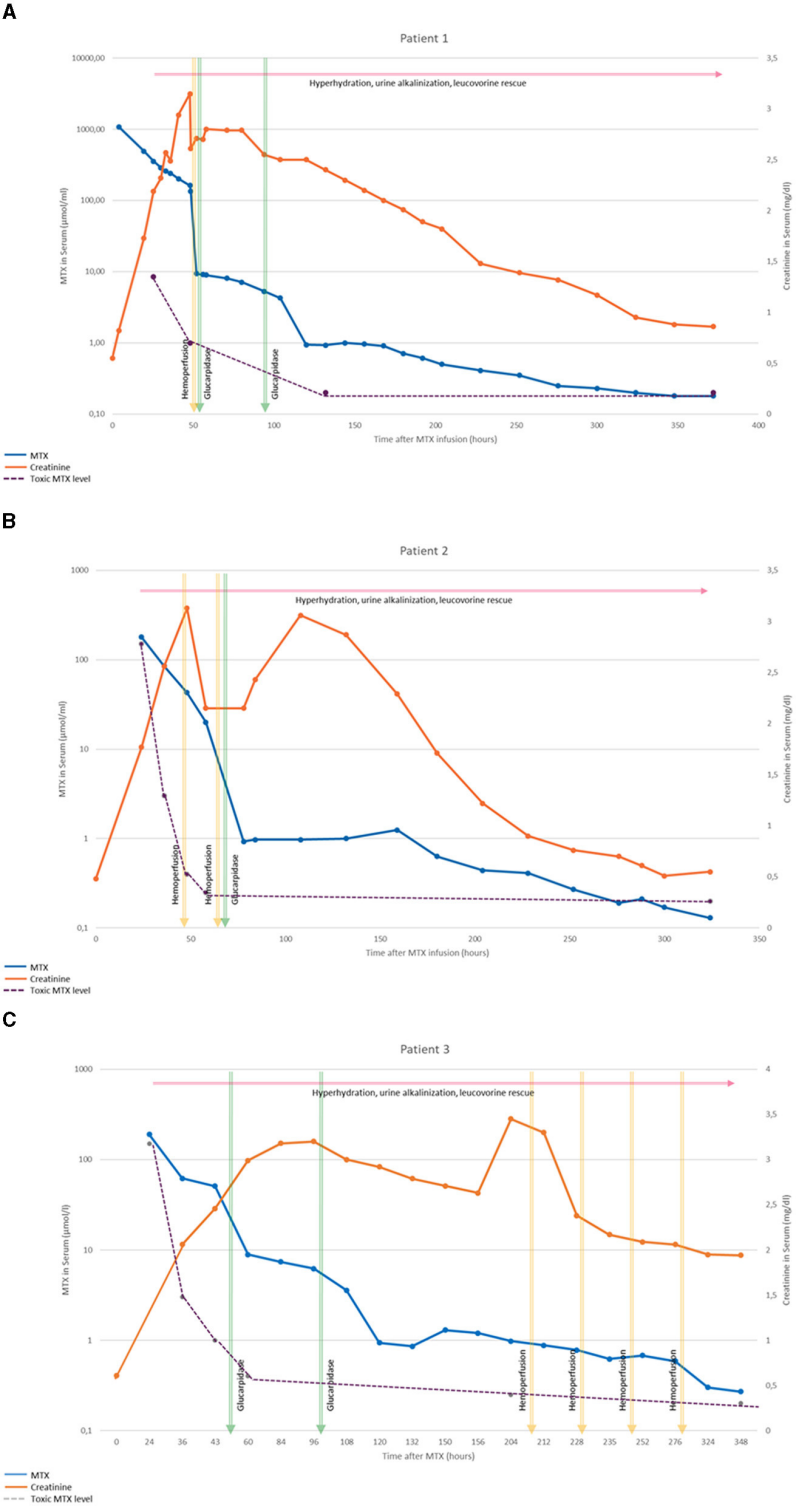
This figure shows the progression of MTX and creatinine levels after HDMTX in the three presented cases [**(A)** patient 1, **(B)** patient 2, and **(C)** patient 3]. Furthermore, the applied supportive treatments are depicted. The dotted line represents the toxic MTX level according to each treatment protocol.

An 11-year-old male (41.1 kg, 151 cm, BSA 1.32 m^2^) treated for second malignant disease (an osteoblastic osteosarcoma involving the facial bones), and previous history of radiotherapy and chemotherapy due to bilateral retinoblastoma during the 1st year of life. He received his first course of HDMTX according to protocol EURAMOS 1 in an external center (EURAMOS 1) (23). Before receiving HDMTX, he showed correct renal and liver function and normal serum albumin. Prehydration (3 L/m^2^) and urine alkalinization were performed as recommended (EURAMOS 1). HDMTX (12 g/m^2^ e.v. over 4 h) was administered with a short interruption due to exanthema during infusion, which improved with antihistaminics and cortisone (MTX at 4 h 1,081 μmol/L, creatinine 0.81). The serum MTX level 24 h after infusion was with 491 μmol/L clearly in toxic range (toxic >8.5 μmol/L, serum creatinine 1.73 mg/dl). An intensification of hyperhydration (5 L/m^2^), forced diuresis (furosemide), urine alkalinization and intensification of treatment with leucovorin (250 mg/m^2^/3 h) were performed. Forty-eight hours after infusion and despite of intensified treatment, MTX levels remained persistently high (202 μmol/L, toxic >1 μmol/L) and the patient developed leukocytosis (34.5 × 10^9^/l), progressive acute renal failure (increase of serum creatinine to 2.94 mg/dL, eGFR 21 ml/min/1.73 m^2^), liver failure with elevated liver enzymes (AST 125 UI/L, ALT 193 UI/L) and coagulopathy (Quick 47%) treated with vitamin K and plasma. Nevertheless, the patient was clinically stable, showed no hypertension, no mucositis and besides erythema and emesis no further symptoms. Due to temporary unavailability of glucarpidase, the patient was referred to our center where one session of CHP was performed at his arrival, as bridge until glucarpidase became available. The first CHP session started 52 h after start of MTX infusion. A significant reduction of MTX level from 202 to 124 μmol/L was achieved using CHP, and the procedure was performed without complications. Further, glucarpidase (50 U/kg) was administered as soon as available (54 h after MTX infusion), which led to a reduction of MTX level to 8.09 μmol/L. Due to persistent elevated MTX levels 96 h after MTX infusion (5.29 μmol/L, toxic >1 μmol/L) the patient received a second glucarpidase dose leading to a sufficient reduction of MTX level. Later on, treatment with leucovorin was maintained until day 31 (MTX <1 μmol/L). Diuresis was preserved and a progressive recovery of renal (creatinine 0.86 mg/dL, eGFR 72 ml/min/1.73 m^2^), normalization of liver function and blood count were observed during the follow-up (Figure 1A).

### Patient 2

A 12 years old male (34.5 kg, 139 cm, BSA 1.15 m^2^) with pre-B lymphoblastic lymphoma, previously healthy and with normal renal function and serum albumin at the start of treatment (creatinine 0,48 mg/dl at 0 h). The patient received the second HDMTX course (5 g/m^2^ e.v., in 24 h), 14 days after first course (which had been well-tolerated), according to Protocol Euro LB 02 (24) after adequate prehydration and urine alkalinization. During the first 24 h after the initiation of MTX infusion the patient developed non-oliguric acute renal failure (creatinine 3.13 mg/dL, eGFR 18 ml/min/1.73 m^2^). An elevated MTX level was observed with 180 μmol/L after 24 h (toxic >150 μmol/L) that was managed by intensification of hydration (4,000 mL/m^2^/day) and leucovorin rescue (100 mg/m^2^/3 h). At 36 h MTX level remained persistently high with 85 μmol/L (toxic > 3 μmol/L) and the patient was referred to our center to start treatment with CHP due the unavailability of glucarpidase. The patient presented clinically stable with abdominal pain and vomiting, epistaxis, no cutaneous lesions, no neurological symptoms, no hypertension. After the first session of CHP (48 h after start of MTX infusion) a decrease of MTX level to 43 μmol/L (toxic > 0.4 μmol/L) was observed, and after the second session an MTX level of 20 μmol/L achieved. Anemia (hemoglobin 6.4 g/dL) and thrombocytopenia (37 × 10^9^/l) were observed at this point, needing blood cell and platelet transfusions. Glucarpidase was administered as soon as available (82 h after start of MTX infusion), decreasing MTX to 0.96 μmol/L. Subsequently, we observed a recovery of renal function (creatinine 0.55 mg/dL, eGFR 104 ml/min/1.73 m^2^) remission of gastrointestinal symptoms and normal blood count (Figure 1B).

### Patient 3

An 11 years old male (46.2 kg, 142 cm, BSA 1.36 m^2^) with T-cell acute lymphoblastic leukemia, under treatment according to protocol ALL SEHOP/PETHEMA 2013 HR (25). The patient had no history of previous diseases, with normal renal and liver function and serum albumin at the start of treatment. During the first course of HDMTX (5 g/m^2^ e.v., in 24 h) with adequate previous hydration and urine alkalinization an elevated MTX level was detected 24 h after starting infusion (190 μmol/L, toxic > 150 μmol/L). Despite adequate treatment with hydration (3,000 ml/m^2^/24 h) and leucovorin rescue (30 mg/m^2^/6 h), the patient presented persistently elevated MTX levels after 36 and 48 h (620 and 510 μmol/L). In addition, non-oliguric renal failure with creatinine increase to 2.36 mg/dL (eGFR 34.5 ml/min/1.73 m^2^) was observed. The patient received the first dose of glucarpidase (50 U/kg) 48 h and second dose 96 h after starting MTX infusion, nevertheless an insufficient decrease in MTX levels was observed (Figure 1). MTX levels continued persistently high (MTX at 100 h 3.8 μmol/L, toxic >0.4) despite two doses of glucarpidase, hydration, urine alkalinization and leucovorin rescue. Glucarpidase was no further available. The patient presented in reduced general status, in the absence of diarrhea, mucositis, or other symptoms such as hypertension. Persistent renal failure (with maintained diuresis) and increasing creatinine (max. creatinine 3.45 mg/dL at day 9 after MTX) was observed at admission. Leukopenia (1.4 × 10^9^/l) was observed too. Rescue treatment with hemoperfusion was indicated. CHP was performed without complications on days 9, 10, 11, and 12 after MTX infusion with no complications other than slight anemia (hemoglobin 8 g/dl) and thrombocytopenia (100 × 10^9^/l). Progressive improvement was achieved under treatment with CHP, with reduction in MTX levels, recovery of renal function and normalization of blood count (Figure 1C).

## Discussion

High-dose methotrexate-induced toxicity is an oncologic emergency that can potentially result in serious organ damage and life threat. The introduction of standardized supportive care measures such as hyperhydration, urine alkalinization, and leucovorin rescue has dramatically reduced the risk of MTX toxicity (6). In the rare cases where pharmacokinetically guided leucovorine rescue is insufficient, glucarpidase has become the treatment of choice (10). However, delays in drug availability may happen. Extracorporeal treatments can be used as a bridge in cases where glucarpidase is not available or insufficiently effective. Studies assessing the efficacy of different extracorporeal treatment approaches for MTX poisoning show mixed results, reporting mostly of isolated cases, presenting no control patients and using differing concomitant interventions.

The election of an extracorporeal treatment method for drug intoxication should rely on the pharmacological characteristics of the toxin, the antagonist availability, and the expertise of the center. Charcoal hemoperfusion (CHP) is based in the adsorption of toxins to charcoal particles, which makes it a suitable method for protein bound toxins, liposoluble toxins and toxins which have high molecular weight, such as MTX. Common side effects are thrombocytopenia, hypocalcemia and hypoglycemia. In the three presented cases CHP was a safe and well-tolerated method leading to a significant reduction in MTX levels.

Despite fast efficacy and improved tolerance with less hypersensitivity reactions and charcoal embolization nowadays, since cellulose coated charcoal filters were introduced, and in parallel with the advance of hemodialysis techniques and availability of high efficacy filters, CHP has lost popularity (11). Decline in the use of CHP relies on the high cost of the columns, which become saturated and loose effectiveness during the treatment, and short expiration time. In contrast to intermittent hemodialysis (iHD), CHP does not contribute to normalize electrolyte or fluid disbalances, and therefore cannot replace acute dialysis if needed (26). Reversely, CHP will cause less electrolyte imbalance in those intoxicated patients with preserved renal function.

Currently, iHD is often used for extracorporeal toxic removal, since it is an effective procedure for a broad range of toxins with low molecular weight and which are water-soluble. However, iHD is less suitable for protein bound toxins depuration, such as MTX. iHD also serves as treatment for fluid and electrolyte disbalances in those patients, who often present acute renal failure. Furthermore, iHD is more frequently available and lower-priced than CHP (26). Reports on the use of iHD in HDMTX toxicity show variable results. Many patients experience a rebound in MTX levels after iHD, probably due to multicompartmental distribution of MTX and slow redistribution from tissues to blood stream (14, 18). Avoiding delays in the initiation of treatment could prevent distribution of MTX in intracellular compartment and maximize removal through extracorporeal treatment (27). High-dose continuous venovenous hemofiltration (CVVHDF) may prevent rebound of plasma drug levels as well (22). Lack of trials comparing CHP vs. iHD efficacy and adverse events in MTX removal does not support an evidenced-based decision process between those procedures.

We report our experience with CHP in MTX toxicity after a regional transient lack of availability of glucarpidase, and as a life-saving treatment in a patient with limited response to the drug. In cases 1 and 2 successful stabilization and reduction of MTX levels were achieved after 1 or 2 sessions of CHP. Glucarpidase was administered as soon as available, leading to a significant reduction of MTX levels. That fast and good outcome indicates that possibly both patients would had responded to glucarpidase if available, but CHP facilitated a fast MTX removal and the achievement of low-risk levels. In comparison, case 3 presented with persistent elevated MTX levels despite 2 doses of glucarpidase, although we have to mention that MTX levels after glucarpidase may be overestimated due to the cross reactivity with its metabolites using an immunoassay for measurement. We could have probably avoided to repeat glucarpidase doses at high cost if a more specific measurement method for MTX would have been available. Treatment with CHP was considered since glucarpidase was no longer available for few days, and the patient remained in a critical situation. Fortunately, under treatment with daily CHP sessions we observed a decrease in MTX levels and normalization of renal function. Unfortunately, leucovorin levels were not measured before and after CHP sessions and could be reduced through CHP leading to insufficient protection during CHP sessions.

Leucovorin rescue was continued in all patients, in parallel to glucarpidase treatment and CHP, since it is the most effective resource to protect cells from MTX toxicity. Leucovorin rescue should be maintained until negative MTX levels are reached as described and replaced after dialysis may be removed through HD and CHP (17). Leucovorin should not be given until 2 h after glucarpidase administration since it could interfere with the cleavage of MTX (28).

Glucarpidase should remain the first line treatment in cases of MTX delayed elimination, even if only insufficient quantity of glucarpidase available, since its efficacy is not dose dependent (29). Recommendations in cases of insufficient decrease in MTX levels after use of glucarpidase are missing. Existing reports refer of no further reduction in MTX levels after extra doses of glucarpidase within 48 h of MTX infusion. The benefit of administering glucarpidase after >60 h can be discussed, since lasting toxicities may not be undone beyond this point (28). In our patients the use of glucarpidase led to further decrease of MTX levels, despite the time of administration. One should consider the possible overestimation of MTX levels after glucarpidase infusion, since most laboratories measure MTX with immunoassay, which shows a cross reactivity with its metabolite DAMPA (30). MTX levels after use of glucarpidase may be overestimated in this report. We could have probably avoid the second dose of glucarpidase and the use of CHP in case 3 if a more specific method for measuring MTX levels, as HPLC, would have been available.

These three cases show that CHP is an eligible bridging rescue treatment approach for life-threatening MTX intoxications in the absence of glucarpidase or after lack of response. Despite limitations such as small sample size, or lack of comparison with iHD, our experience shows a fast and positive response to few CHP sessions in severe cases with MTX toxicity, in the absence of significant adverse events or technical issues, but venous central access requirement. Acute renal failure was successfully managed by conservative treatment in all cases, and renal function recovered spontaneously in parallel to MTX level reduction.

Therefore, and based on MTX body distribution, we favor the use of CHP instead of iHD as recue treatment of refractory MTX toxicity. CHP does not replace supportive measures or rescue with leucovorin but should be considered in centers with expertise in the method and available cartridges since it leads to a significant reduction of MTX levels with concomitant improvement of renal function.

Up-to-date treatment recommendations for MTX toxicity are urgently needed. The international EXTRIP (extracorporeal treatments in poisoning workgroup) reviewed the use of extracorporeal treatments for MTX toxicity recently, a report on the results should be available soon. Furthermore, we retrospectively evaluated the course of MTX levels in our patients using the MTXPK.org tool, which is very practical and easy to use resource for clinical decision making (31).

## Data Availability Statement

The data analyzed in this study is subject to the following licenses/restrictions: Data out of patients' clinical records. Requests to access these datasets should be directed to alejandra.rosales@i-med.ac.at.

## Ethics Statement

Ethical review and approval was not required for the study on human participants in accordance with the local legislation and institutional requirements. Written informed consent from the participants' legal guardian/next of kin was not required to participate in this study in accordance with the national legislation and the institutional requirements.

## Author Contributions

AR analyzed and interpreted the data, drafted the article, and approved the final version. AM, MM, and JD provided content of critical importance, revised the article, and approved the final version. GA conceived the study, drafted and revised the article, provided content of critical importance, and approved the final version. All authors contributed to the article and approved the submitted version.

## Conflict of Interest

The authors declare that the research was conducted in the absence of any commercial or financial relationships that could be construed as a potential conflict of interest.

## Publisher's Note

All claims expressed in this article are solely those of the authors and do not necessarily represent those of their affiliated organizations, or those of the publisher, the editors and the reviewers. Any product that may be evaluated in this article, or claim that may be made by its manufacturer, is not guaranteed or endorsed by the publisher.
